# Congenital anterior polar cataract associated with a missense mutation in the human alpha crystallin gene *CRYAA*

**Published:** 2011-10-15

**Authors:** Lu Zhang, Yi Zhang, Ping Liu, Wenping Cao, Xianling Tang, Sheng Su

**Affiliations:** Eye Hospital, the First Affiliated Hospital, Harbin Medical University, Harbin, China

## Abstract

**Purpose:**

To identify the potential pathogenic mutation over four generations of a Chinese family with congenital anterior polar cataracts (APC).

**Methods:**

We investigated four generations of a Chinese family who are afflicted with anterior polar cataracts. The family resides in a relatively isolated region of Northern China. Peripheral blood samples were collected from all of the family members, and genomic DNA was then extracted from the blood samples. A gene scan was performed using about 400 primers labeled with fluorescent stain. Linkage software defined the region of the diseased gene with a Linkage analysis, and Cyrillic software processed the resulting haplotypes. Mutation detection was performed in the candidate gene by sequencing amplified products.

**Results:**

A maximum logarithm of odds score (LOD) score was obtained at marker D21S1252(LOD score [Z]=3.23, recombination fraction [θ]=0.0. Haplotype analysis traced the disease gene to an 18.47 cM region bounded by D21S263 and D21S266 on chromosome21q22.11-q22.3. Direct sequencing of the candidate alpha A crystallin (CRYAA) gene revealed a c.347G>A transition in exon 3 of *CRYAA* that co-segregated with the cataract in the family members and was not observed in 100 control patients. This single-nucleotide change resulted in the substitution of a highly conserved Arginine by Histidine (R116H).

**Conclusions:**

The present study identified a missense mutation (R116H) in the *CRYAA* gene that causes autosomal dominant congenital anterior polar cataracts in a Chinese family. Our finding confirms the high rate of apparently independent mutations at this dinucleotide.

## Introduction

A congenital cataract is a clinically and genetically heterogeneous lens disorder that typically appears as a sight-threatening trait during childhood, accounting for one-tenth of the cases of childhood blindness [1]. Approximately half of all congenital cataract cases are inherited either in isolation or as part of a syndrome of ocular or systemic abnormalities [2]. All three classical forms of Mendelian inheritance have been associated with non-syndromic cataracts. However, the majority of families with a history of congenital cataracts show an autosomal dominant transmission pattern.

Crystallins are separated into two classes: taxon-specific (or enzyme) and ubiquitous. The latter class, which is at issue here, constitutes the major proteins of a vertebrate eye lens and maintains the transparency and refractive index of the lens. Since lens central fiber cells lose their nuclei during development, these crystallins are made and then retained throughout life, requiring that they be extremely stable proteins. Mammalian lens crystallins are divided into alpha, beta, and gamma families. Beta and gamma crystallins create a superfamily. Alpha and beta families, however, are further divided into acidic and basic groups. Seven protein regions exist in beta-crystallins: four homologous motifs, a connecting peptide, and NH_2_- and COOH-terminal extensions. Gamma-crystallins have the same regions without the terminal extensions. Alpha crystallins are composed of two gene products: alpha-A and alpha-B, for acidic and basic, respectively.

Alpha crystallins can be induced by heat shock and, as such, are considered members of the small heat shock protein family. They act as molecular chaperones although they do not renature proteins and release them in the fashion of a true chaperone; instead the alpha crystallins hold the proteins in large soluble aggregates. Alpha crystallins are also an autokinase activity and participate in intracellular architecture. Alpha-A and alpha-B gene products are differentially expressed; alpha-A is preferentially restricted to the lens.

In this study, we identified the genetic defect causing autosomal dominant congenital anterior polar cataracts over four generations of a Chinese family.

## Methods

### Clinical evaluation and DNA specimens

**Figure 1:**
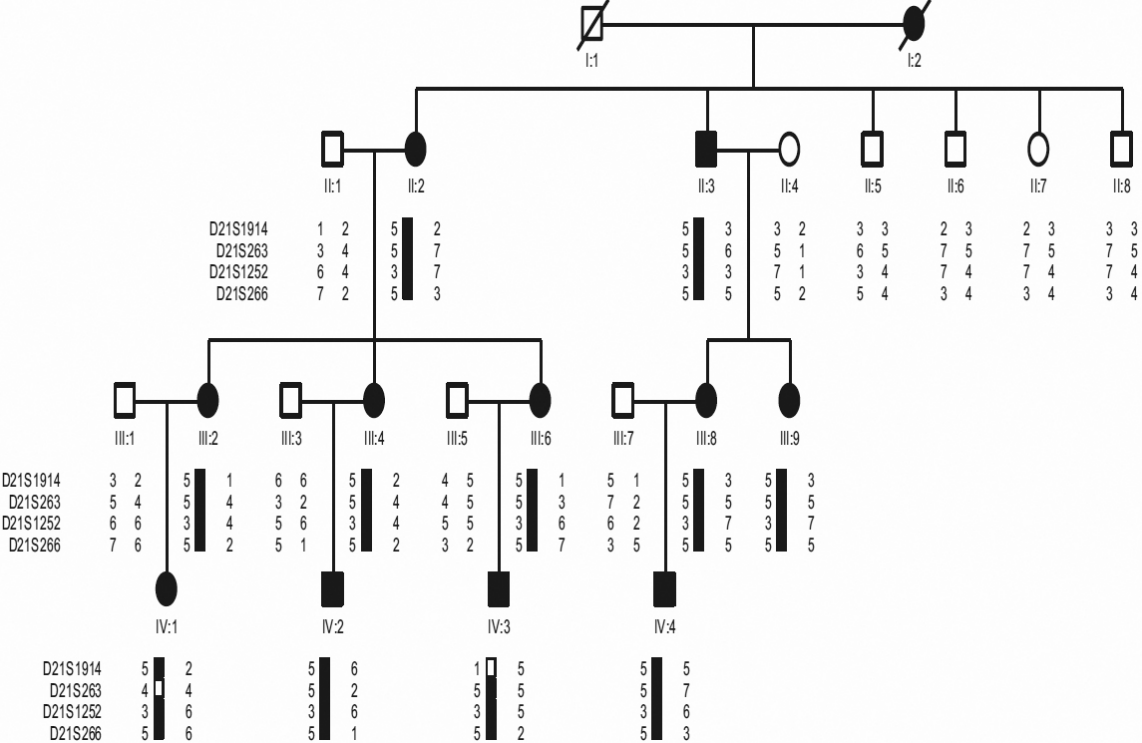
Pedigree and haplotype of the cataract family.

We identified four generations of a Chinese family with autosomal dominant anterior polar cataract in the absence of other ocular or systemic abnormalities. The family resides in a relatively isolated region of Northern China. All participants provided informed consent in accordance with the Declaration of Helsinki. Twenty-one family members participated in the study, eleven affected and ten unaffected (Figure 1). Family members were considered “affected” by a history of cataract extraction or ophthalmologic examination, which included visual acuity testing, slit lamp examination, intraocular pressure measurement, and fundus examination with dilated pupils. Phenotypes were documented using slit lamp photography. Peripheral blood was collected and genomic DNA was extracted from blood leukocytes using a QIAampDNA Blood Mini Kit (Qiagen, Hilden, Germany).

### Genotyping and linkage analysis

We conducted a genome wide linkage scan based on a set of dinucleotide repeat microsatellite markers spaced at approximately 10 cM intervals using an ABI PRISM Linkage Mapping Set Version 2.5 (Applied Biosystems, Foster City, CA). A “touchdown” polymerase chain reaction (PCR) was performed in a 5 μl reaction volume containing 20 ng of genomic DNA, 1µl of 10× PCR buffer, 7mM MgCl_2_, 0.2mM dNTPs, 0.3 U HotStar Taq DNA polymerase, and 0.05µM microsatellite markers. After an initial denaturation period of 12 min at 95 °C, 14 cycles were performed at 95 °C for 30 s, 63–56 °C for 30 s (with a 0.5 °C decrease at each step), and 72 °C for 1 min. Thirty cycles were performed at 95 °C for 30 s, 56 °C for 30 s, and 72 °C for 1 min followed by an extension at 72 °C for 10 min and a final hold at 4 °C. The PCR products were pooled on the basis of size (Genescan-400HD ROX; Perkin Elmer, Foster City, CA), denatured at 95 °C for 1 min, and electrophoresed in a 96 capillary automated DNA analysis system (MegaBACE 1000; Amersham, Freiburg, Germany). The results were analyzed by Genetic Profiler, Version 1.5 (Amersham). Two point logarithm of odds score (LOD) scores (Z) were calculated using the LINKAGE software package (Version 5.1). A gene frequency of 0.0001 and a 100% penetration were assumed for the cataract locus. LOD scores were calculated at recombination fractions (θ) of 0.00, 0.1, 0.2, 0.3, 0.4, and 0.5.

### Mutational analyses

One functional candidate gene (alpha A crystallin [*CRYAA*]) is within the physical region defined for the cataract on 21q22.11-q22.3. We systematically sequenced *CRYAA*, which contains three exons and two introns, in two affected and two unaffected members of the family using specific primers (Table 1). Genomic DNA was PCR amplified, purified, and sequenced directly using dye-terminator chemistry. The purified PCR products were sequenced on both DNA strands using an ABI 3100 sequencer (Applied Biosystems). After identifying a missense mutation in exon 3 of *CRYAA*, all of the family members and 100 unrelated normal individuals were screened.

**Table 1:** PCR primers for mutational screening of *CRYAA*.

**Exon**	**Strand**	**sequence (5′-3′)**
1	Sense	CCTTAATGCCTCCATTCTGC
** **	Antisense	AGCAAGACCAGAGTCCATCG
2	Sense	GGTGACCGAAGCATCTCTGT
** **	Antisense	GTCCCTCTCCCAGGGTTG
3	Sense	CCCCCTTCTGCAGTCAGT
** **	Antisense	GGGAAGCAAAGGAAGACAGA

## Results

### Clinical findings

**Figure 2:**
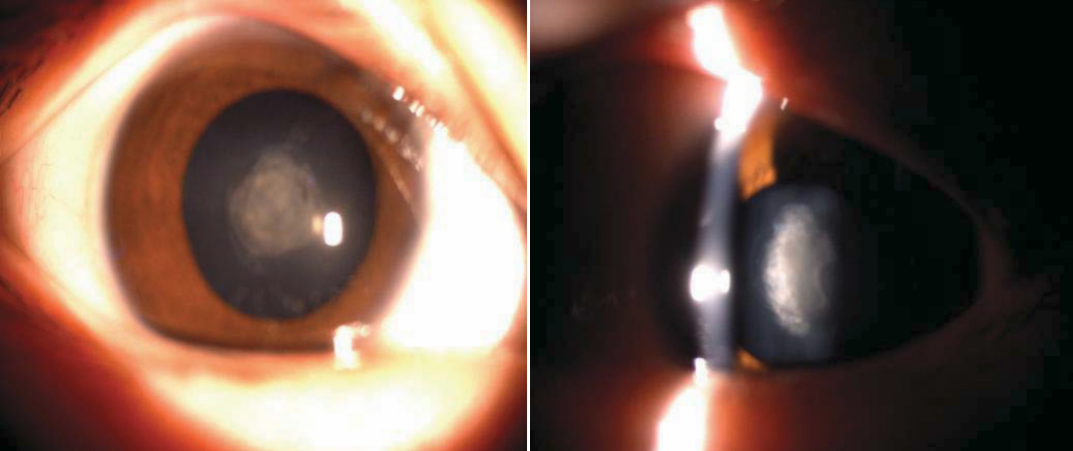
Slit lamp photographs of the eye of affected individual IV:2.

After reviewing the family’s data, we found that autosomal dominant inheritance of the cataract phenotype was supported by the presence of affected individuals in each of the four generations and male-to-male transmission. The affected individuals presented with bilateral congenital anterior polar cataract (Figure 2). The opacities varied in size from <1 to >4 mm in diameter and were bilateral in all. The smaller opacities were flat while the larger opacities were raised and laminated to form a pyramidal cataract protruding from the anterior pole of the lens along the optical axis. The rest of the lens was clear, although in the larger opacities the anterior cortex was minimally affected.

We also found that a cataract was present at birth. All affected individuals had the same poor visual acuity of hand movement in front of the eye. They had nystagmus and amblyopia regardless of the size of the opacity. There was no evidence of anterior lenticonus (symptomatic of Alport syndrome) and no family history of other ocular or systemic abnormalities.

### Linkage analysis

By following the exclusion of large chromosomal regions, we obtained suggestive evidence of linkage for marker D21S266 (Z=2.93, θ=0). Further analysis of the marker showed a positive LOD score and strongly supported this candidate region. The maximum two-point LOD score (Z_max_) of 3.23 was obtained at marker D21S1252, where recombination θ=0.00. The results of the two-point LOD scores are summarized in Table 2.

**Table 2:** Two-point LOD scores for linkage analyses.

** **	** **	**Lod score at θ=**							
**Markers**	**Position (cM)**	**0.00**	**0.1**	**0.2**	**0.3**	**0.4**	**0.5**	**Z_max_**	**θ_max_**
D21S1914	19.39	−0.17	2.20	1.79	1.18	0.46	0.00	2.20	0.1
D21S263	27.40	−2.01	0.69	0.67	0.48	0.20	0.00	0.69	0.1
D21S1252	35.45	3.23	2.64	1.99	1.26	0.52	0.00	3.23	0.00
D21S266	45.87	2.93	2.39	1.78	1.12	0.44	0.00	2.93	0.00

### Haplotype analysis

We constructed haplotypes of the family. The markers used are listed in Table 2. Figure 1 shows haplotype data. A crossover between D21S1914 and D21S263 in individual IV:3 and D21S263 and D21S1252 in individual IV:1 defined the proximal border of the region. Marker D21S266, the last marker of chromosomal 21, defined the distal border. The diseased haplotype shared by all affected members was identified. The results of both the linkage and haplotype analyses situated the diseased gene in a 18.47 cM region bounded by D21S263 and D21S266 at 21q22.11-q22.3.

All affected individuals had an affected parent, and none of the unaffected individuals carried the diseased haplotype. Thus, penetrance appears to be virtually complete in this family.

### Mutational analysis

**Figure 3:**
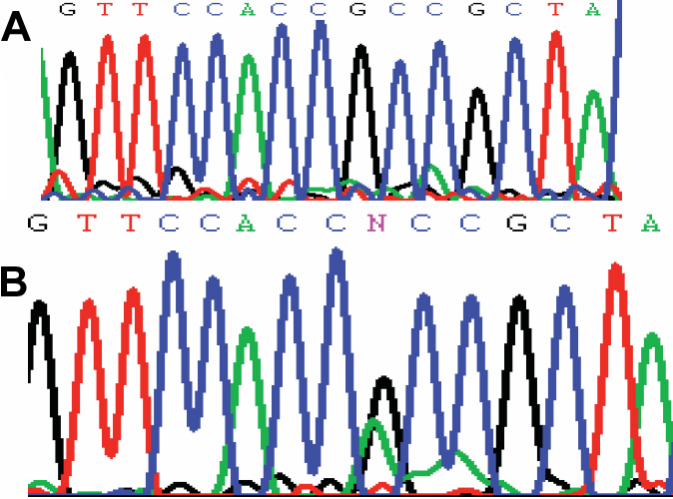
Mutational analysis of *CRYAA*. **A**: Sequence chromatograms of the wild-type *CRYAA* allele, showing that the wild-type gene encodes a arginine residue (CGC). **B**: Sequence chromatograms of the mutant allele, showing a G→A transition that substituted a histidine residue (CAC).

Sequence analysis of *CRYAA* identified a c.347G>A transition in exon 3. This single-nucleotide change was predicted to result in a missense mutation at codon 116, changing a phylogenetically conserved Arginine residue to Histidine residue (R116H). The co-segregation of the c. 347G>A transition was only found in affected family members and was absent both in the unaffected family members and the control group. This result strongly suggests that the R116H substitution was a causative mutation rather than a benign single nucleotide polymorphism (SNP) in linkage disequilibrium with the cataract (Figure 3).

## Discussion

Anterior polar cataracts (APC), which are small opacities on the anterior surface of the lens, usually do not interfere with vision. This distinctive cataract phenotype is also associated with certain other genetic eye diseases including Aniridia and Peter's anomaly and may present in certain systemic diseases (e.g., Alport syndrome). APC can occur as an autosomal dominant, autosomal recessive, or X-linked trait [3]. The prevalence of APC among all cataracts was high in Korea in comparison with any other country [4] with a rate of 6.02% during the five months of the hospital-based study. In addition, eighty-seven percent of patients with APC were male. Anterior polar cataracts are relatively uncommon, but have been described in association with mutations in EPH receptor A2 (*EPHA2*), alpha 8 gap junction protein (*GJA8*), gamma D crystallin (*CRYGD*), eyes absent homolog 1 (*EYA1*), major intrinsic protein of lens fiber (*AQP0*), heat shock transcription factor 4 (*HSF4*), and beta B1crystallin (*CRYBB1*). They are also a fairly common result of mutations in *CRYAA*, including the one described here [5].

To our knowledge, APC pedigree is rare. APC has been shown to segregate with an unbalanced 3;18 chromosome translocation [6] and a balanced reciprocal 2;14 chromosomal translocation [3]. In the 3;18 translocation, a cataract was associated with dysmorphic features presumably resulting from partial trisomy of 3q and partial monosomy of 18p. The 2;14 translocation, however, suggests that a gene for APC lies near the breakpoints at 2p25 or 14q24. By using genetic linkage analysis with microsatellite markers for four generations of an English family, Berry et al. [7] identified a locus for an autosomal dominant anterior polar cataract on 17p12–13.

In addition to the R116H mutation, there are three other mutations in *CRYAA* that have been associated with cataracts:

Litt et al. [8] found that a R116C mutation is associated with an autosomal dominant congenital zonular central nuclear cataract. Vanita et al. [9] also identified a R116C mutation causing fan-shaped cataract-microcornea syndrome in an Indian family;
Pras et al. [10] identified a W9X mutation in *CRYAA* causing autosomal recessive cataract in an inbred Jewish Persian family; and
Mackay et al. [11] identified a R49C mutation in *CRYAA* that underlies an autosomal dominant form of “nuclear” cataract segregating in four generations of a Caucasian family.

Interestingly, the same mutation in R116H had recently been suggested to cause the polymorphic cataract, microcornea, or corneal opacity in a Chilean family [12] and the polymorphic cataract in a Chinese family [13]. Both the polymorphic cataract in a Chilean family, which was a diverse and novel cataract with variable morphology (anterior polar, cortical, embryonal, fan-shaped, or anterior subcapsular), and the polymorphic cataract in a Chinese family, which showed three cataract phenotypes: punctuate, nuclear, and total cataracts, were clinically different from our isolated anterior polar cataract.

Recent functional studies have shown that the missense mutation R116H resulted in an altered size distribution, impaired packing of the secondary structures, and modified quaternary structure with great hydrophobic exposure [14]. The mutant exhibited a substrate-dependent chaperone (aggregation–inhibition) or anti-chaperone (aggregation–promotion) effect. Equilibrium unfolding experiments indicated that the mutation stabilized an aggregation-prone intermediate that was not populated during the unfolding of the wild-type protein. The accumulation of this intermediate greatly promoted the formation of non-native large oligomers or aggregates during unfolding. These results suggested that both the aggregation of the mutant upon stress and co-deposition with the target proteins were likely to be responsible for the onset of cataracts.

In conclusion, we report the identification of a misense mutation (R116H) in *CRYAA* that co-segregated with autosomal dominant congenital anterior polar cataract in four generations of a Chinese family. Our finding confirms the high rate of apparently independent mutations at this dinucleotide.
